# Intravascular large B‐cell lymphoma treated with polatuzumab‐based salvage therapy: A rare case

**DOI:** 10.1002/jha2.301

**Published:** 2021-09-21

**Authors:** George Bitar, Andrew Wotherspoon, Ayoma D. Attygalle, David Cunningham, Bhupinder Sharma

**Affiliations:** ^1^ The Royal Marsden Hospital London London UK; ^2^ The Institute of Cancer Research London UK

An 84‐year‐old gentleman presented with fatigue, appetite and weight loss. Positron emission tomography/computed tomography (PET/CT) demonstrated ^18^fluorodeoxyglucose (FDG)‐avid adrenal (left image) and rectal masses. Intravascular large B‐cell lymphoma (IVLBCL) was diagnosed from CT‐guided adrenal biopsy. Morphologically there was a proliferation of large atypical lymphoid cells (middle panel, haematoxylin and eosin; 20× objective) expressing CD20 (top right; 10× objective). The intravascular localisation of atypical B cells was highlighted by the endothelial marker CD34 (bottom right; 10× objective). Neoplastic B cells expressed CD19, CD22, CD79a, MUM‐1/IRF4, bcl‐6 and bcl‐2 protein and were negative for CD3, CD5, CD10, CD23, cyclinD1, CD30, TdT and EBER, with MYC over‐expression and Ki 67 proliferation index of almost 100%. Bone marrow (BM) was not involved at diagnosis. Rectal biopsy revealed adenocarcinoma (no evidence of lymphoma).

Following six cycles of rituximab, gemcitabine, vincristine, cyclophosphamide and prednisolone (R‐GCVP) and two cycles of intravenous methotrexate (for central nervous system prophylaxis), PET/CT revealed metabolic complete response (mCR) of lymphoma; however, rectal tumour progression with biopsy proven liver metastases. Post‐treatment for metastatic rectal disease, biopsy of new liver and cutaneous lesions, and BM demonstrated IVLBCL relapse (now CD5 positive). Six cycles of rituximab, polatuzumab vedotin (PV), and bendamustine led to a mCR. A subsequent PET/CT demonstrating FDG‐avid non‐size significant nodes was interpreted as relapse. On specialist review, this was considered to reflect a Deauville score of ‘X’ (‘activity which may be unrelated to lymphoma") and possibly reactive to Covid‐19 vaccination (IVLBCL usually spares lymph nodes). Rituximab treatment was continued, however the patient relapsed with extensive Stage IV disease (PET/CT right image) including numerous extranodal sites of involvement at 3 months post polatuzumab salvage. This also demonstrates the aggressive natural history of IVLBCL and the need for effective consolidation therapy.


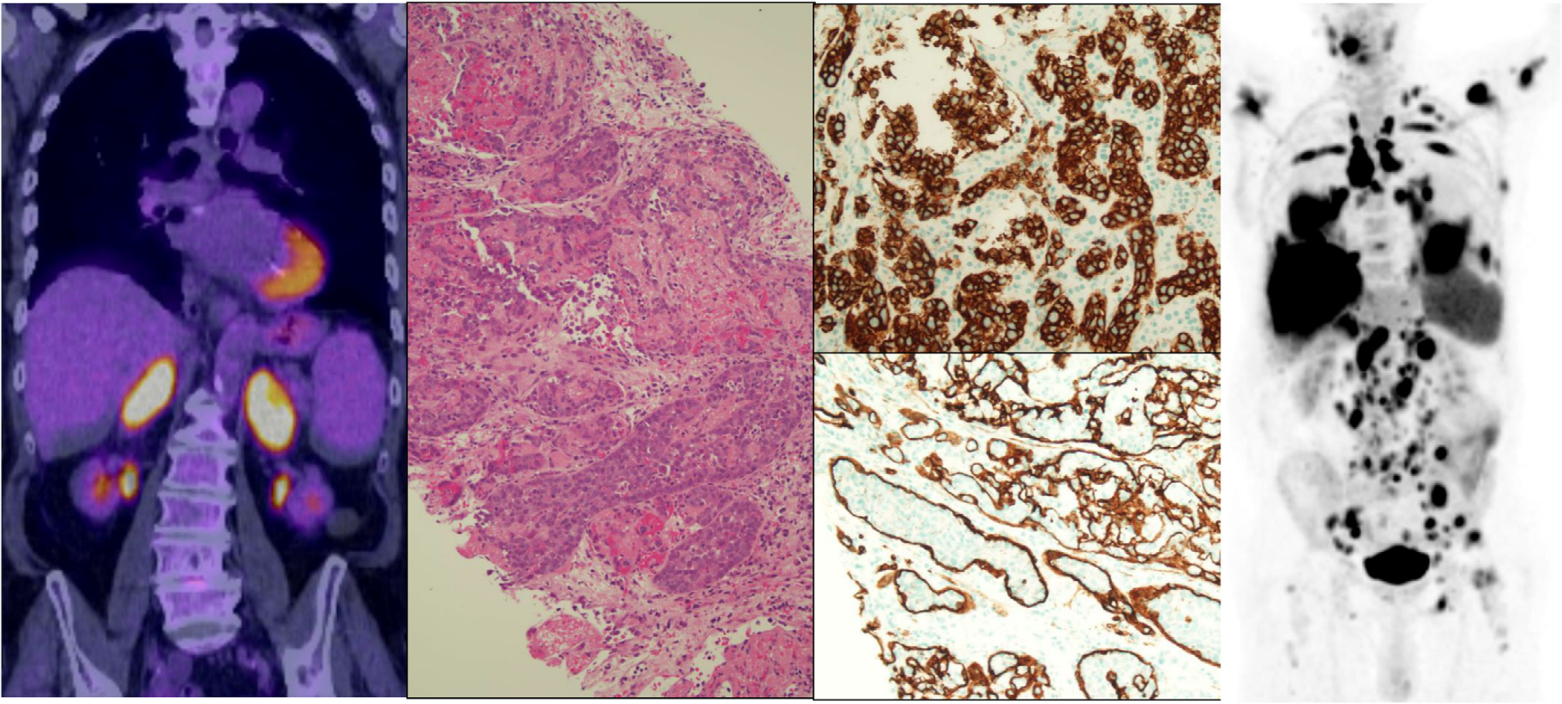


IVLBCL is a rare extranodal large B‐cell lymphoma characterised by selective growth of lymphoma cells within vessel lumina, usually widely disseminated in extranodal sites (including the bone marrow). Two main types occur: classic disease with symptoms related to the main organ involved; a haemophagocytic syndrome‐associated disease with multi‐organ failure, hepatosplenomegaly and pancytopenia (both poor prognosis); an isolated cutaneous variant (better prognosis) is also recognized. To our knowledge, this is only the second published case for use of PV in IVLBCL.

